# Not the norm: Face likeness is not the same as similarity to familiar face prototypes

**DOI:** 10.1177/20416695231171355

**Published:** 2023-05-02

**Authors:** Benjamin Balas, Adam Sandford, Kay Ritchie

**Affiliations:** Psychology Department, North Dakota State University, Fargo, ND, USA; Psychology Department, University of Guelph-Humber, Toronto, Ontario, Canada; School of Psychology, 4547 University of Lincoln, Lincoln, UK

**Keywords:** face perception, social cognition, familarity, face likeness

## Abstract

Face images depicting the same individual can differ substantially from one another. Ecological variation in pose, expression, lighting, and other sources of appearance variability complicates the recognition and matching of unfamiliar faces, but acquired familiarity leads to the ability to cope with these challenges. Among the many ways that face of the same individual can vary, some images are judged to be better *likenesses* of familiar individuals than others. Simply put, these images look more like the individual under consideration than others. But what does it mean for an image to be a better likeness than another? Does likeness entail typicality, or is it something distinct from this? We examined the relationship between the likeness of face images and the similarity of those images to average images of target individuals using a set of famous faces selected for reciprocal familiarity/unfamiliarity across US and UK participants. We found that though likeness judgments are correlated with similarity-to-prototype judgments made by both familiar and unfamiliar participants, this correlation was smaller than the correlation between similarity judgments made by different participant groups. This implies that while familiarity weakens the relationship between likeness and similarity-to-prototype judgments, it does not change similarity-to-prototype judgments to the same degree.

Images of any individual's face can be highly variable. Changes in viewing conditions such as lighting, viewpoint, and distance (Burton et al., 2010; Noyes & Jenkins, 2017) can induce substantial image-to-image variability, and appearance also varies a great deal due to changes in expression, age, and other intrinsic factors (Burton et al., 2016; Jenkins et al., 2011; Fysh & Bindemann, 2018; Redfern & Benton, 2017). This within-person variability poses substantial difficulties for the matching and discrimination of unfamiliar face images. In particular, it has been established across multiple studies that within-person variability in appearance leads observers who are unfamiliar with an individual to presume that images depicting the same person are more likely to belong to different individuals (Jenkins et al., 2011), a phenomenon that has been described as a failure to “tell people together.” (Andrews et al., 2015; Balas et al., 2019). Familiarity with an individual essentially erases these difficulties, leading even highly variable images to be reliably matched or recognized as belonging to the same person (Dowsett et al., 2016). It, therefore, appears that there are not generalizable lessons to be learned about how to cope with within-person face variability, but rather that one must become familiar with individual faces to achieve robust recognition performance subject to appearance variation. Besides conferring the ability to cope with variability, familiarity also supports the use of idiosyncratic variability as its own signature of identity, allowing observers who are familiar with a face to use specific variation in appearance as a means of recognition (Burton et al., 2016; Kramer et al., 2017). Within-person variability thus remains a critically important aspect of facial appearance to understand more fully, especially with regard to face learning (Bruce 1994; Burton, 2013; Young & Burton, 2017).

One way in which images of the same individual vary from one another is in terms of a property called *likeness*. Colloquially, likeness refers to the extent to which an image of an individual looks like that person. A bad likeness does not reflect the typical appearance of an individual while a good likeness does. Dan Llewellyn Hall's portrait of Queen Elizabeth II elicited much criticism due to its perceived poor representation of the monarch's appearance, for example, while Lucian Freud's 2000 portrait drew a more mixed response, in part due to what some referred to as its “honest” depiction of the Queen's countenance. Few of us will have cause to be concerned with how well a commissioned portrait conveys our own likeness, but likeness judgments are nonetheless pervasive: Casual photos, identifying documents, and any other images of ourselves all vary in terms of how well they can be said to look like us. Images judged to be good likeness are also processed differently by the visual system. Ritchie et al. (2018) demonstrated a number of interesting properties of “good likeness” images, including the observation that images rated to be good likenesses were recognized more quickly and also that increased familiarity with an individual increased likeness ratings across variable images. They also report that likeness ratings are idiosyncratic, however, suggesting that personal experience with faces matters a great deal in establishing what likeness means for a particular face, while still having measurable perceptual consequences. This corroborates an earlier finding that different participants declared different images as being a bad likeness of the same familiar individual (Hay et al., 1991). These results suggest that perceived likeness is a meaningful consequence of acquired familiarity with an individual, reflecting specific expertise for that individual's appearance (Hancock, 2021).

Likeness judgments, familiarity, and face learning are likely tightly linked. Ritchie et al. (2018) found that familiarity with an individual was correlated with likeness judgments of highly variable images of that person's face. Therefore, as we learn what a person looks like, we become more tolerant of their variability. In fact, previous research has shown that exposure to variability is helpful for face learning (e.g., Murphy et al., 2015; Ritchie & Burton, 2017). Therefore, increased exposure to variability leads to better learning or higher familiarity with a face, which is characterized by tolerance of variability, giving rise to higher likeness judgments of variable face images.

Likeness has been used as a measure of manipulation strength or success in a variety of studies, such as those investigating distinctiveness (e.g., Lee & Perrett, 2000), caricaturing effects (e.g., Rhodes et al., 1987), and face composites (e.g., Bruce et al., 2002). None of these studies, however, have defined precisely what the term “likeness” means or how it is judged. The closest definition in the literature is that likeness must be a judgment comparing the target image to a stored representation of the person pictured, and that this is an idiosyncratic judgment based on the observer's prior experience with the person pictured (Ritchie et al., 2018). This definition, therefore, requires that the observer be familiar with the person pictured to judge likeness, as an unfamiliar observer would not have a stored representation with which to compare the target image. This raises the important theoretical issue of what exactly this stored representation is. It has often been suggested that we may store familiar people as a prototype or average comprising multiple instances of that person (Burton et al., 2005). Average faces are recognized with exceptional accuracy by machines (Jenkins & Burton, 2008, 2011; Ritchie et al., 2018; Robertson et al., 2015) and tend to be recognized more quickly by human observers. Evidence for the accuracy of human recognition from face averages is, however, mixed. Although studies on unfamiliar face matching (i.e., the ability to determine whether two images show the same person) have not shown a consistent improvement with face averages (Ritchie et al., 2018, 2020; c.f. White et al., 2014), studies using familiar faces show good recognition from averages (Burton et al., 2005). It is possible, therefore, that likeness judgments of familiar faces are really an estimate of how close a particular image is to that individual face average or norm.

In the current study, we chose to address this specific question about the nature of face likeness: What is the relationship between likeness judgments and prototypicality? Bailly and Balas (2019) assumed that this relationship might hold in a study of face learning, demonstrating the effects of similarity-to-prototype on subsequent performance in a training task designed to teach observers new face identities. Although likeness seems commensurate with several of the properties of prototypicality (as operationalized via average faces) described above, there are also some reasons to question whether or not one is a reasonable substitute for the other. For example, the idiosyncrasy of likeness judgments as observed by Ritchie et al. (2018) is somewhat at odds with likeness being closely linked to averageness. Face averages are relatively stable after approximately 10–15 instances are averaged together (Bulthoff & Zhao, 2021), so different experience with an individual is unlikely to yield an estimate of their average appearance that differs a great deal from another person's estimate. It is also not clear that face images rated as good likenesses all look similar to each other—intuitively, photographs depicting a characteristic expression or some other distinct aspect of a person's appearance may strike us as good likenesses of that individual. The likeness may not just be about capturing what you look like on average but also about what makes you look different from other people. Indeed, Hayes. (2020) reported that familiar observers tended to emphasize facial distinctiveness over face typicality when assessing the likeness of portraits and that lower morphological accuracy was related to higher likeness ratings (Hayes et al., 2018). Thus, there is an unresolved relationship between likeness and prototypicality, particularly with regard to face averages that merits direct investigation.

To measure the relationships between familiarity, likeness ratings, and similarity-to-prototype judgments, we recruited two samples of participants from the US and the UK. The advantage of working with these distinct groups of participants is that it allowed us to identify sets of mutually unfamiliar celebrities for each group, such that US participants tended to be familiar with US celebrities and unfamiliar with UK celebrities and vice versa. This allowed us to collect likeness and familiarity ratings from familiar observers and similarity-to-prototype ratings from familiar *and* unfamiliar observers. We suggest that this is critically important because it is altogether possible that familiarity may influence both likeness evaluations and similarity-to-prototype judgments, so including a set of unfamiliar similarity-to-prototype judgments in our analysis makes it possible for us to compare likeness both to familiar prototypicality and unfamiliar prototypicality. If likeness is indeed closely related to average face appearance, likeness ratings should be highly correlated with similarity-to-prototype judgments, each of which would be positively related to familiarity. Alternatively, weak correlations between these variables would indicate that likeness judgments are to some extent independent of prototypicality judgments, incorporating other aspects of facial appearance, and potentially specific types of appearance variability.

## Methods

### Participants

We recruited two groups of participants, one sample (*N*  =  73 Mean age  =  19 years, [min. 18 years, max. 24 years], 46 female, 26 male, 1 nonbinary participant) of US participants and another of UK participants (*N*  =  73, Mean age  =  32 years [min. 18 years, max. 67 years], 55 female, 17 male, 1 participant identifying as “other”). The former group of participants was recruited using the North Dakota State University Undergraduate Psychology study pool, and the latter was recruited by student researchers at the University of Lincoln. All participants provided informed consent before completing the study. This final sample represents participants who indicated that they were familiar with at least two of the celebrities depicted in their population's group of familiar faces. Participants who indicated that they were familiar with one or zero of these individuals were not tested further. Besides this, no other screening criteria were used to recruit and enroll participants. Recruitment and testing procedures were approved by both the North Dakota State University IRB and the University of Lincoln's Research Ethics Committee.

### Stimuli

We conducted an initial pilot survey to identify celebrities from the US and UK that were typically familiar to viewers from the same country and unfamiliar to viewers from the other country. We identified 18 US and 12 UK celebrities and surveyed 28 US and 32 UK participants (demographic information was not collected for these participants). Participants were given a list of celebrity names and were asked simply to indicate which celebrities they were familiar with. From these familiarity rates, we selected identities that were recognized by no more than 10% of the “unfamiliar” participants, and by over 80% of the “familiar” participants. This left us with seven UK celebrities (four male, three female), and we picked the seven US celebrities who best fitted the recognition criteria outlined above (five male, two female).

**Figure 1.**
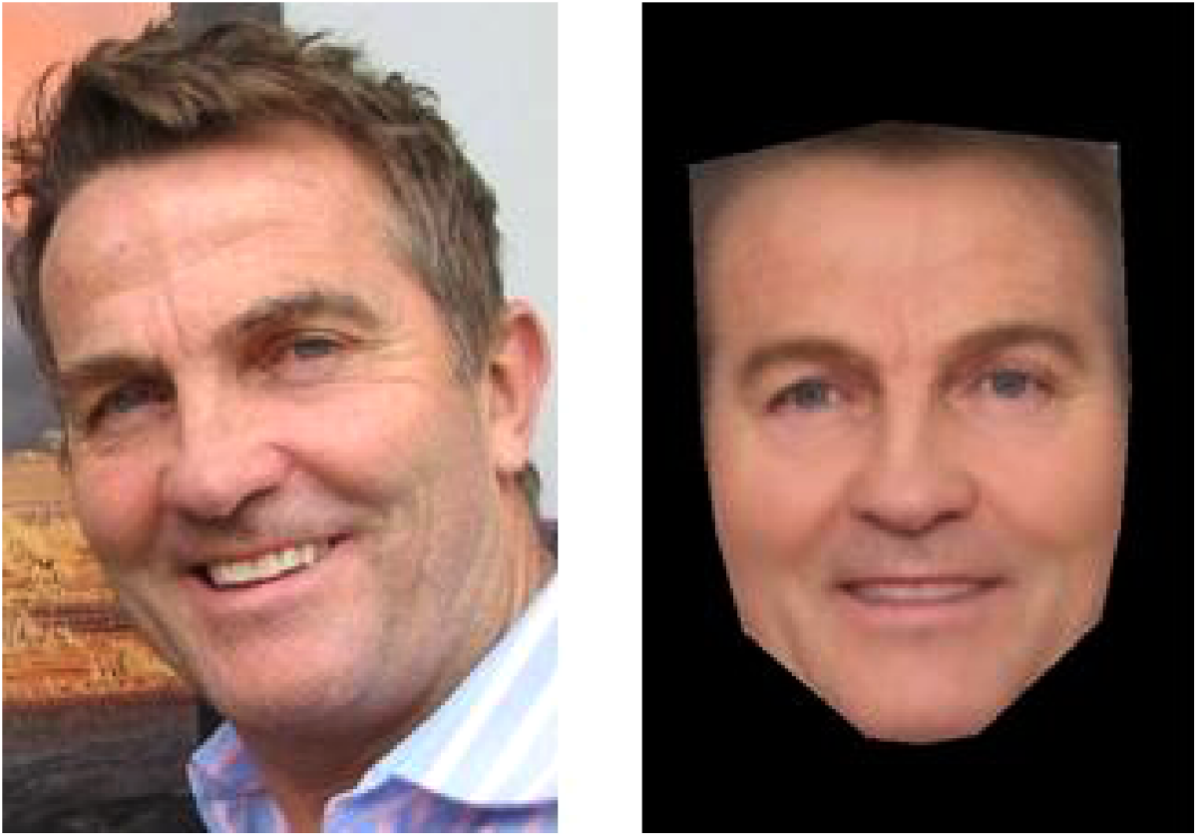
Exemplar and average image of UK celebrity, Bradley Walsh. Exemplar image labeled for reuse (Sully1989, CC BY-SA 3.0 <https://creativecommons.org/licenses/by-sa/3.0>, via Wikimedia Commons), average image created from 30 exemplars using InterFace (Kramer et al., 2017).

Next, we obtained 30 unique images of each celebrity using ordinary image search tools, ensuring that each image: (1) Depicted a frontal view of the individual such that both ears were visible, (2) Was free of sunglasses, hats, or other external occluding elements, and (3) Was in full color. These images were cropped to a final size of 380  ×  570 pixels, retaining the full external contour of the face and head. We also generated an average image of each individual by morphing together the 30 images described above (see Figure 1 for example stimuli). The morphing operation was performed using the InterFace software package (Kramer et al., 2017) “landmarking tool” and “averaging tool.” The landmarking tool obtains each face image's shape by registering 82 points on the face aligned to anatomical features. The averaging tool then morphs each of the 30 images of an identity to their images’ average shape and calculates the mean RGB color values for each pixel, creating a face average or prototype.

### Design and Procedure

All participants completed all tasks in the same order. Participants were first shown a list of all of the “familiar” celebrities (celebrities from their country) and were asked to indicate whether they were familiar with them. The instruction here read “Below is a list of celebrities’ names. Please select all of the celebrities you are familiar with (i.e., people who you would recognize if you saw a picture of them).” This allowed us to only show participants images of celebrities from their country with whom they were actually familiar. Participants in each group then completed a *likeness* task and a *similarity-to-prototype* task. The *likeness* task was only completed using images of familiar celebrities (US participants did not complete this task with UK celebrities and vice-versa). The *similarity-to-prototype* task was completed with both familiar and unfamiliar celebrities in separate blocks.

The *likeness* task required participants to rate each image of a familiar celebrity on a 1–7 Likert scale according to the perceived quality of that image's likeness (1  =  Very poor likeness, 7  =  Very good likeness). The images were blocked by identity and images were randomized within identity blocks. The order of identities was randomized across participants.

The *similarity-to-prototype* task by contrast required participants to view each celebrity image paired with the average image of the same individual and rate how similar the target image was to the average on a 1–7 Likert scale (1 = Not at all similar, 7  =  Very similar). Participants were given unlimited time to make each judgment. Again the images were blocked by identity and images were randomized within identity blocks. The order of identities was randomized across participants.

Finally, to confirm that participants were indeed familiar and unfamiliar as intended with each set of celebrity images, we also asked participants to rate their familiarity with each individual based on celebrity names alone. Altogether, participants tended to complete these tasks in approximately 30 min (average across all participants), though this varied across participants as a function of the number of familiar celebrities identified (SD ∼ 5.75 min).

## Results

### Likeness Ratings and Familiarity

US participants reported recognizing a mean of 3.41 US celebrities, and their mean familiarity rating for these celebrities was 5.50. UK participants reported recognizing a mean of 5.30 UK celebrities, and their mean familiarity rating of 6.21. We compared the number of recognized celebrities and familiarity ratings between participant groups using two independent samples t-tests. UK participants both recognized more of their “familiar” celebrities than US participants *t*(144)  =  7.21, *p* < .001, Cohen's *d*  =  1.19 and rated those celebrities as more familiar than US participants *t*(144)  =  3.10, *p*  =  .002, Cohen's *d*  =  0.51.

In prior work (Ritchie et al., 2018), increased familiarity with an individual led to overall higher likeness ratings across images. That is, more familiarity with a person means more images of that person seem like good likenesses. We examined this relationship in our data by calculating for each participant a per-celebrity mean likeness rating, yielding between 2 and 7 values determined by how many of their country's celebrities that participant was familiar with—for example, a participant who was only familiar with four of the seven target celebrities would only contribute those 4 mean likeness values to our analysis along with the accompanying familiarity ratings for those four celebrities. Next, we examined the correlation between this mean likeness rating per celebrity and participants’ rated familiarity with each celebrity. This analysis yielded different results in the two participant groups: The relationship between familiarity and likeness ratings did not reach significance in the US sample (Spearman's rho  =  0.094, *p*  =  .16) but we observed a significant positive correlation in the UK sample (Spearman's rho  =  0.29, *p* < .001).

### Likeness Ratings to Exemplars as Compared to Prototypes

A simple way to examine the extent to which similarity-to-prototype is a reasonable proxy for likeness is to determine how likeness judgments are made to prototypical faces as compared to individual exemplars that contribute to that prototype. For each participant, we averaged together their likeness ratings for each celebrity's exemplar images (*N*  =  30 per celebrity), then averaged these values together to obtain an aggregate exemplar likeness rating. Next, we calculated the average of each participant's likeness ratings for the prototype images of each celebrity to obtain an average prototype likeness rating. We used a paired-sample t-test to determine whether the average prototype likeness rating differed significantly from the average exemplar likeness rating across participants for both US and UK participants. For US participants, we found that the mean likeness rating to exemplars (*M*  =  4.99, *SEM*  =  0.13) was significantly higher than the mean likeness rating assigned to prototypes (*M*  =  2.52, *SEM*  =  0.16; *t*(71)  =  15.08, *p* < .001, Cohen's *d*  =  1.78). We observed the same pattern in the UK data—mean likeness ratings to exemplars (*M*  =  4.66, *SEM*  =  0.15) were significantly larger than likeness ratings to prototypes (*M*  =  4.33, *SEM*  =  0.11; *t*(73)  =  3.23, *p*  =  .0019, Cohen's *d*  =  0.38). Although we do find an effect in the same direction in both groups of participants, the size of this effect clearly differs. As all aspects of face averaging were the same across these groups of participants and stimuli, we do not have a strong hypothesis as to why this would be the case.

### Likeness and Similarity-to-Prototype Correlations

**Figure 2.**
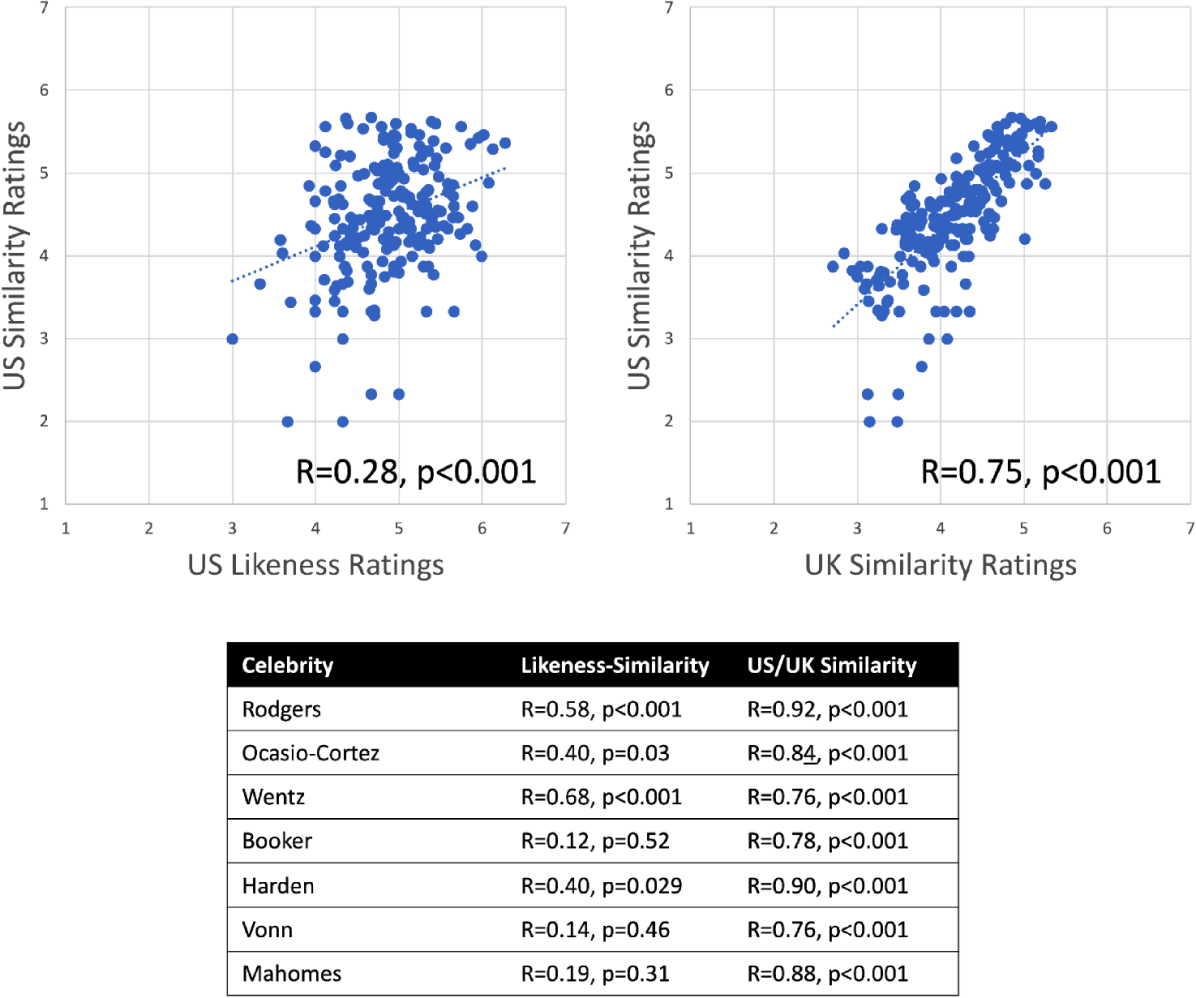
Omnibus correlations for US celebrities: At top left, we display the scatterplot for *Likeness* ratings vs. *Similarity-to-Prototype* ratings made by US participants. At top right, we display the scatterplot for *Similarity-to-Prototype ratings* made by US participants vs. those made by UK participants. Below, we report these correlations for each US celebrity.

**Figure 3.**
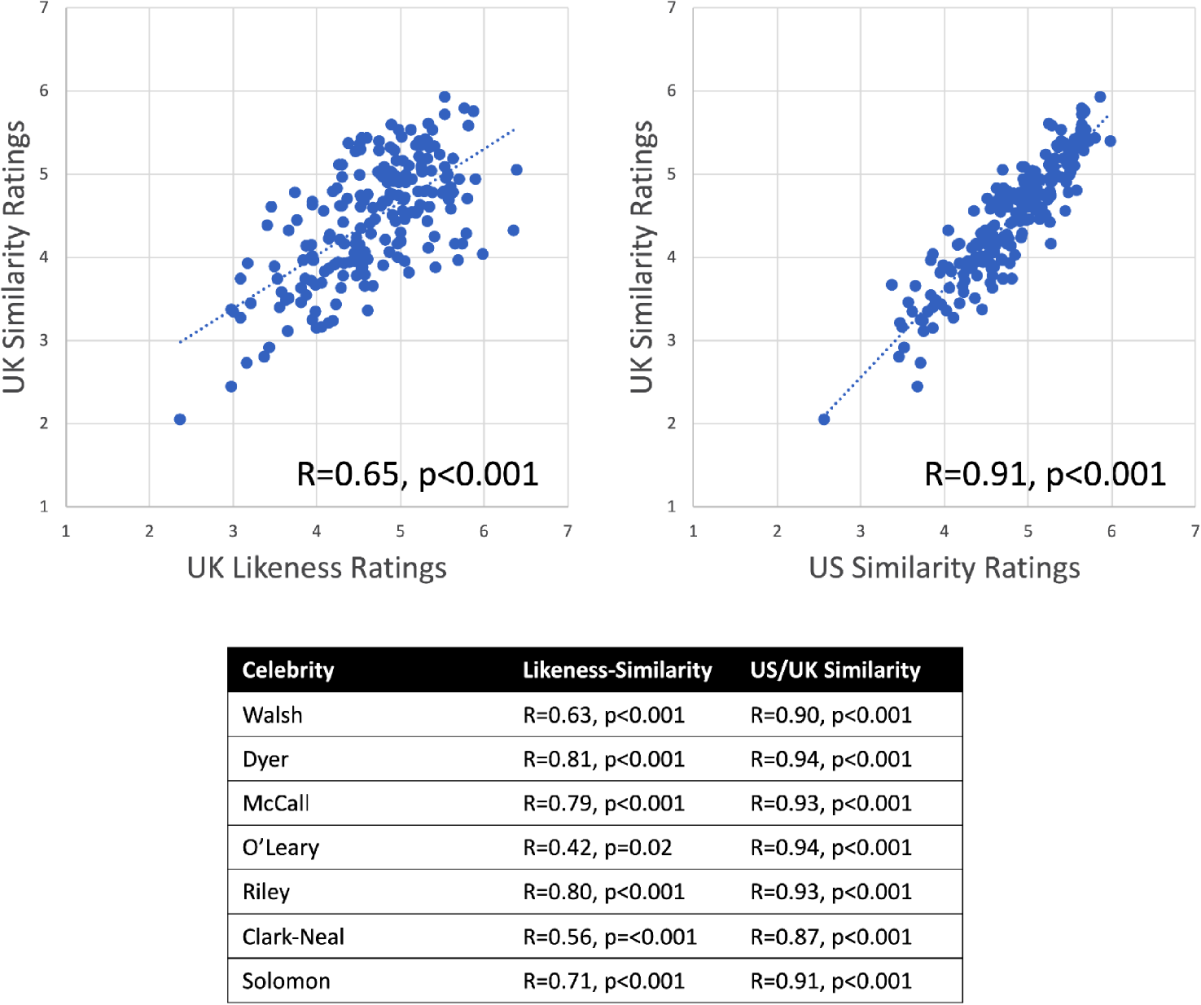
Omnibus correlations for UK celebrities: At top left, we display the scatterplot for *Likeness* ratings vs. *Similarity-to-Prototype* ratings made by UK participants. At top right, we display the scatterplot for *Similarity-to-Prototype ratings* made by UK participants vs. those made by US participants. Below, we report these correlations for each UK celebrity.

To examine the extent to which likeness is adequately captured by the similarity of each image to a prototype, we calculated the mean likeness rating assigned to each familiar image across participants as well as the average similarity-to-prototype rating for each familiar and unfamiliar image. On a per-image basis, how closely is likeness related to similarity-to-prototype? We carried out omnibus correlations for the US (Figure 2) and UK data (Figure 3) separately, yielding correlation coefficients for *Familiar Likeness vs. Familiar Similarity-to-Prototype* and *Unfamiliar Similarity-to-Prototype vs. Familiar Similarity-to-Prototype*.

We find that all of these omnibus correlations reached significance—*likeness* correlated positively with *similarity-to-prototype,* and *similarity-to-prototype* judgments made by familiar and unfamiliar participants also correlated positively. In both datasets, we also observed that the correlation between *likeness* and *similarity-to-prototype* judgments was smaller than the correlation between the two sets of *similarity-to-prototype* judgments. We assessed this by carrying out a bootstrap analysis to estimate 95% confidence intervals for each correlation coefficient, which revealed that for both groups of participants, the correlation coefficient observed between similarity-prototype estimates was indeed meaningfully larger than that observed between likeness and similarity-to-prototype judgments. Specifically, for US observers the 95% CI for the correlation between likeness and similarity-to-prototype judgments was [0.21 0.46] which does not overlap with the 95% CI of [0.69 0.81] observed for the correlation between similarity judgments across participant groups. Likewise, the 95% CI for the correlation between likeness and similarity-prototype judgments was [0.56 0.72], which also does not overlap with the 95% CI of [0.88 0.93] for the correlation between similarity judgments. At the level of individual celebrities, this pattern was also fairly consistent, though several celebrities did not yield positive correlations between likeness and similarity-to-prototype judgments after correcting for multiple comparisons.

## Discussion

Our data reveal several aspects of likeness and its relationship to identity prototypes. First, we partially replicated Ritchie et al. (2018) result concerning familiarity and likeness: We found, as they did, that increased familiarity with an individual leads to overall higher ratings of likeness across images of that person, but only in our UK population. Although likeness is not an especially meaningful concept in the absence of familiarity, this result shows that familiarity effects can be dose-dependent. More familiarity with a person has a continued influence on how images of that individual are recognized and perceived. We also found that face averages yielded lower likeness scores (though with different effect sizes across our UK and US participants—a difference we did not expect), in keeping with prior results demonstrating the same (Ritchie et al., 2018). By itself, the replication of this data point is an argument against a particularly close relationship between likeness and similarity to face prototypes. We would expect that an average face created from combining a few dozen images of a particular person should reflect an identity norm well, yet these are consistently evaluated as looking less like the person than some of the constituent images that contribute to the average. Likeness already appears to be something distinct from prototypicality or averageness, but perhaps this also depends on the manner in which prototypicality is evaluated as a function of acquired familiarity.

Our data collected from participants who were mutually unfamiliar with faces from the home country of the other group is of particular value here. By comparing results across samples, we are able to examine the robustness of similarity-to-prototype judgments when familiarity differs vastly across participant groups and also compare that relationship to the coupling of likeness to prototypicality judgments. We found that similarity-to-prototype judgments were very stable across participant groups, which means that familiarity does not confer a particular advantage or distinct mode of processing faces that affects these judgments. This result is not just useful in terms of establishing the reliability of our measure (though this is indeed valuable) but helps to dissociate familiarity from prototypicality judgments, making them a more meaningful comparison variable both within and between participant samples when we consider how likeness was evaluated. We found that while there were positive correlations between likeness and similarity-to-prototype judgments, these were not nearly as strong as those between prototypicality judgments across participant groups. This was the case regardless of which participant group's similarity-to-prototype judgments we used for examining likeness, largely due to the close agreement between groups about prototypicality. We take this to mean that while likeness clearly has some relationship to prototypicality, it is not correct to use either variable as a proxy for the other. More directly, likeness judgments are not simply another expression of prototypicality, at least when considered with regard to an average face.

What then *is* a likeness judgment? Likeness has something to do with perceptual fluency, perhaps something to do with distinctiveness given the lack of a strong relationship with typicality, and certainly, something to do with familiarity, but what exactly ties these various results (ours and others) together? Our data do not offer many constraints on a model of likeness perception, but one possibility that we think is worth considering further is that likeness may reflect the structure of more complex summaries of familiar face appearance than a standard norm-based description. Many face-space models of recognition assume that exemplars of each individual are used to estimate an average appearance (norm, prototype, etc.) and some region of acceptable variability around that average. This has been expressed in terms of “attractor fields” (Tanaka et al., 1998) or in probabilistic models that use such distributions to estimate likelihoods for identification (Moon & Phillips, 2001). A common feature of many such models is that they are unimodal: There is an assumption that an individual's appearance is captured effectively by a single average and an estimate of variation around that. When one examines how images of the same face are distributed in different computational spaces, however, it is evident that this is not necessarily borne out. Tenebaum et al. (Tenenbaum et al., 2000) demonstrate in their ISOMAP model that many objects with a small number of degrees of freedom that govern appearance (the rigid rotation of a head in 3D for example) yield images that are constrained to lie on a curved manifold, for example. Recent explorations of the structure of face identity within hierarchical layers of deep neural networks trained for identification have also revealed curvilinear manifolds like these that reflect parametric variation in illumination and viewpoint (O’Toole et al., 2018). Such structures can be challenging to learn so that subsequent inferences may be based on them, but may more closely capture the variation associated with different exemplars of the same individual's face. Critically, they are not well described by an average point and the spread around that average.

What does this have to do with likeness and prototypicality? We suggest that increased familiarity with an individual may lead not just to a better estimate of a unimodal structure for their appearance variability, but to the estimation of a more complex structure. Specifically, whether or not variability in facial appearance is best captured by a curving manifold or something blobbier, we suggest that increased familiarity may lead to multiple norms or prototypes being estimated for a single individual. That is, identification would not proceed by comparing a new image to a single norm and determining whether the variation in question is acceptable, but instead by asking whether or not a new exemplar is sufficiently close to any of the multiple norms we have established for a familiar face. We suggest that in this framework, likeness may indeed be an expression of prototypicality *but only if one knows what the multiple norms are for each person*. In the current study, the single average we estimated from the images we collected per celebrity may have yielded low likeness values and been a poor proxy for likeness judgments in general because familiarity led to a fragmented estimate of typical appearance per individual, none of the parts of which were close to that global average. Moreover, establishing multiple norms per person may also help explain why likeness estimates increase or improve with familiarity: The better one gets at estimating the complex structure associated with one individual's appearance variability, the closer new exemplars will tend to be to one of the multiple norms you have established. This is in some ways a proposal that familiar face recognition is characterized neither by a unimodal norm-based model nor a norm-free exemplar model, but by a sort of hybrid structure that includes multiple linked estimates of typical appearance and variation around those points in face space. Such a model is consistent with retained image-specific details of familiar and unfamiliar faces (Dunn et al., 2019) though perhaps by different mechanisms. To our knowledge, the possibility that familiarity may lead observers to establish multiple average images of a face has not been explored, but we think our results perhaps offer a good reason to test this hypothesis.

Our data are not without some important limitations, however. Most conspicuously, the data from our US participants is not nearly as clear-cut as our data from UK participants. For example, the per-celebrity relationship between familiarity and likeness was not observed for US participants, perhaps owing to the comparatively large number of missing trials and missing celebrities on the basis of self-reported familiarity. Regardless, however, even considering the UK data largely in isolation and only using US estimates of similarity-to-prototype for UK celebrities yields interesting and useful data consistent with our discussion above. Besides these differences across participant groups, we also acknowledge that including a wider range and larger number of celebrity, faces would undoubtedly be valuable. Presently we sought to balance our desire for a reasonably large number of images per celebrity against the constraints of an online testing session, but are thus limited in terms of the scope of unique individuals presented here. Ideally, it would be particularly interesting to examine these issues in the context of personal face familiarity as there are many results demonstrating that familiarity effects are often especially robust when images of individuals known to the participants can be used (Ritchie et al., 2015). Practically, this presents a number of substantial challenges, of course, but would be an interesting avenue for further work examining the relationships between these variables.

A limitation with this type of task in general is that likeness ratings and similarity-to-prototype judgments inherently rely on different processes. In order to judge an image for likeness, the viewer must compare the image on screen to an abstract representation in their mind. Similarity-to-prototype judgments, on the other hand, are a simple perceptual comparison of two on-screen stimuli. If we assume that abstracted representations of familiar people are akin to a prototype, then what we have tried to achieve here is to present the abstraction (the prototype) alongside each individual image. It is, however, difficult to reconcile the differences between the two tasks, which may ultimately have led to the different strengths of correlations observed between the tasks.

Overall, our results demonstrate what likeness likely is not and perhaps provide some interesting hints of what it might be. Further, our data support and extend prior results regarding the role of familiarity in likeness and prototypicality judgments. These results are a useful foundation for exploring further issues related to the nature of face likeness and the manner in which highly variable exemplars of an individual's face ultimately contribute to the successful learning of their identity for the purposes of recognition.
